# Desire for Parenthood in Context of Other Life Aspirations Among Lesbian, Gay, and Heterosexual Young Adults

**DOI:** 10.3389/fpsyg.2019.02679

**Published:** 2019-11-28

**Authors:** Doyle P. Tate, Charlotte J. Patterson

**Affiliations:** Department of Psychology, University of Virginia, Charlottesville, VA, United States

**Keywords:** sexual orientation, parenthood, LGBTQ, parenting desires and intentions, aspirations (psychology)

## Abstract

Research has established that sexual minority young adults generally report fewer desires and fewer expectations for parenthood than do their heterosexual peers. Little is known, however, about other desires and expectations. Is parenthood the only domain in which lesbian and gay individuals report fewer desires and expectations than their heterosexual peers? Or do lower aspirations among lesbian and gay adults about parenthood also occur in other domains, such as marriage and work? In this study, we explored a variety of desires and expectations for the future among lesbian, gay, and heterosexual young adults. Participants for this internet survey were recruited via social media, and included 368 childless cisgender young adults (211 lesbian or gay and 157 heterosexual) living in the United States. There were three main findings. First, while lesbian/gay individuals were less likely than heterosexual participants to express desire for parenthood, desires in the other future domains did not vary across sexual orientation. Lesbian/gay participants were as likely as heterosexual individuals to desire marriage, friendships, and community connections, as well as career and economic success. Results for expectations were, however, very different. Lesbian/gay participants were less likely than heterosexual individuals to expect that they would marry, become parents, feel connected to a community, achieve meaningful careers, live in their ideal housing, or that they would attain financial stability. Thus, although desires were largely unrelated to sexual orientation, many expectations were strongly linked to it. Lesbian and gay individuals were also far more likely than their heterosexual peers to desire future goals that they did not expect to achieve. Overall, for lesbian/gay young adults, low parenthood aspirations were part of a general pattern of low expectations (though not reduced desires) across a number of life domains.

## Introduction

Research has established that lesbian and gay individuals report lower aspirations for parenthood than do heterosexual individuals (Patterson and Riskind, 2010; Riskind and Patterson, 2010; Baiocco and Laghi, 2013; Riskind and Tornello, 2017; Jeffries et al., 2019; Leal et al., 2019; Shenkman et al., 2019; Tate et al., 2019). As we use the term here, parenthood aspirations include: (1) parenthood desires, i.e., how much people want to become parents, (2) parenthood expectations, i.e., how likely people think they are to become parents, and (3) parenthood intentions, i.e., whether people are planning to pursue parenthood (Gato et al., 2017; Tate and Patterson, 2019a). Lesbian and gay individuals in the United States (U.S.) report lower parenthood desires, expectations, and intentions than do heterosexual individuals (Riskind and Tornello, 2017; Tate and Patterson, 2019a). Little is known, however, about the generality of this finding. Is parenthood the only domain in which lesbian and gay individuals report fewer desires and expectations than their heterosexual peers? Or do lower desires and expectations among lesbian and gay adults about parenthood also extend to other domains, such as marriage and career? Furthermore, gaps between aspirations for parenthood as a function of sexual orientation have been found (Shenkman, 2012; Riskind and Tornello, 2017), but little is known about how desires and expectations may or may not coincide for other life goals as a function of sexual orientation. In this study, we explored a variety of future desires and expectations among lesbian, gay, and heterosexual young adults.

Lesbian and gay individuals typically have lower parenthood aspirations than heterosexual individuals, and scholars have studied reasons for this finding (Goldberg et al., 2007, 2012; Robinson and Brewster, 2014; Simon et al., 2018; Scandurra et al., 2019; Shenkman et al., 2019; Tate et al., 2019). Lesbian and gay people, both in the U.S. and abroad, face many more societal barriers that limit their access to parenthood than do most heterosexual individuals (Berkowitz and Marsiglio, 2007; Goldberg et al., 2007, 2012; Robinson and Brewster, 2014; Blake et al., 2017; Scandurra et al., 2019; Leal et al., 2019). Moreover, lesbian and gay individuals typically have more strained social relationships, particularly with their parents, than do heterosexual individuals, and this has been found to partially explain disparities in parenthood intentions (Tate et al., 2019). Similarly, Simon et al. (2018) found that lesbian women expressed a greater preference to be employed full-time and in a permanent position before pursing parenthood than did heterosexual or bisexual women (Simon et al., 2018). In addition, Tate and Patterson (2019a) examined perceptions of social and economic costs involved with parenthood, and found that lesbian women report higher costs than did heterosexual women. Lesbian women’s higher perceived costs explained some of the observed differences in parenthood desires, expectations, and intentions as a function of sexual orientation (Tate and Patterson, 2019a).

Moreover, some authors have reported discrepancies in family formation aspirations as a function of sexual orientation, particularly for men (Shenkman, 2012; Riskind and Tornello, 2017). For instance, Riskind and Tornello (2017) found that 20% of gay men reported desire for parenthood, but no intention to pursue parenthood. This difference occurred in only 5% of heterosexual men, 10% of heterosexual women, and 9% of lesbian women, and the majority of these groups reported both desires and intentions to pursue parenthood (Riskind and Tornello, 2017). In addition, Shenkman (2012) found that while 68% of sexual minority men in Israel reported strongly desiring parenthood, only 31% of those men expected to become parents. This finding also extended to couplehood, with 91% of single men strongly desiring couplehood, but only 43% of those men expecting to find a meaningful relationship (Shenkman, 2012). Thus, there is evidence, at least for sexual minority men, that gaps between family formation desires and expectations may exist.

There has been some work to suggest that lesbian, gay, and heterosexual youth envision their futures in similar ways, particularly when it comes to marriage and parenthood (D’Augelli et al., 2008). Little is known, however, about how findings about future family formation might extend to other aspects of the future, such as finding a meaningful career or achieving financial stability. Frazier and Hooker (2006) reviewed how young adults envision their futures, and found that many young adults hope first to achieve educational goals, find ideal occupations, then marry, start families, and achieve financial success (Frazier and Hooker, 2006). In addition to these goals, many young adults hope to take on civic responsibilities, to find a congenial group of friends, and to manage a home (Hooker et al., 1996). Young adults also reported that they would be concerned if they did not achieve these goals, particularly personal, family, and material goals (Hooker et al., 1996; Frazier and Hooker, 2006).

When envisioning their future lives, lesbian and gay individuals may have more uncertainties than their heterosexual peers. Lesbian and gay individuals often experience heterosexist and gender harassment at work and can be fired from their jobs based on their sexual orientation in much of the U.S. (Rabelo and Cortina, 2014). Moreover, many states do not have housing protections for lesbian and gay individuals, and sexual minority individuals face housing discrimination throughout much of the country (Quartey, 2018). Hate crimes against lesbian and gay individuals have also sharply risen since 2016 (National Coalition of Anti-Violence Programs, 2018). Moreover, research has found that lesbian and gay individuals have less supportive family relationships than heterosexual individuals (Patterson et al., 2018; Tate and Patterson, 2019b). Thus, it seems possible that lesbian and gay individuals might have different expectations than their heterosexual peers about their future prospects.

This study explored desires and expectations for the future among lesbian, gay, and heterosexual young adults, with three main research questions:

(1) How do desires for the future differ as a function of sexual orientation?
(2) How do expectations for the future differ as a function of sexual orientation?
(3) Are there differential gaps between future desires and expectations as a function of sexual orientation?

Based on earlier findings, we expected that desires for parenthood would be greater among heterosexual individuals than among lesbian and gay individuals (Riskind and Tornello, 2017; Tate and Patterson, 2019a). We expected that lesbian and gay individuals would report lower expectations for parenthood and other life achievements than would heterosexual individuals (Tate and Patterson, 2019a; Tate et al., 2019). Based on previous work (Shenkman, 2012; Riskind and Tornello, 2017), we expected that substantial gaps would exist between desires and expectations for the future, especially for lesbian and gay individuals.

## Materials and Methods

### Participants

Participants were 368 childless cisgender individuals from the U.S. who were recruited via social media, including Facebook, Reddit, Twitter, and email listservs, and participated in an online survey. The data were collected over 2 months in the fall of 2018. The eligibility conditions for this survey were that individuals live in the U.S., be between 18 and 35 years of age, and have no children. The ages of 18–35 years were selected because we were interested in how early and young adults envision their futures, and we used measures designed for this age group (Frazier and Hooker, 2006). The sample was comprised of cisgender childless lesbian, gay, and heterosexual individuals (53 lesbian women, 158 gay men, 97 heterosexual women, and 60 heterosexual men). Sexual orientation was assessed via individuals’ self-report about sexual identity. Participants were asked which of the following identities best fit them, “Heterosexual,” “Bisexual,” “Pansexual,” “Lesbian/Gay,” “Asexual,” “Other: Please Specify.” Only heterosexual, gay, and lesbian cisgender individuals were included in this study due to small sample sizes for other identities, especially among men. This study was approved by the Social and Behavioral Sciences Institutional Review board (IRB) at our institution.

### Measures

#### Demographics

We assessed several demographic characteristics (Table 1). Age was self-reported by participants (in years). Race/ethnicity was coded into three categories: White (*n* = 244, 66%), multiracial (*n* = 43, 12%), and single-race racial/ethnic minority (*n* = 81, 22%). The single-race racial/ethnic minority category consisted of 27 (33%) Latino/Latina/Latinx participants, 25 (31%) individuals of East or South Asian descent, 24 (30%) African American individuals, and 4 (5%) people of other racial/ethnic identities. Education was assessed using a scale of 1 = “Less than a high school degree” to 8 = “Professional degree (MD or JD).” Finally, romantic relationship permanence was assessed by asking individuals the degree to which they thought that their relationships (if any) were permanent. Single people were coded as 0 and those in relationships were coded as 1 = “Almost no chance” to 5 = “Almost 100% chance.”

**TABLE 1:** Differences in demographic variables as a function of sexual orientation.

	**Sexual orientation (S.O)**							
	**Heterosexual**		**Gay/Lesbian**					
**Demographics *n* =**	**Male (60)**	**Female (97)**	**Male (158)**	**Female (53)**	**Test statistic_S.O_**	**Test statistic_Gender_**	**Test statistic_Interaction_**	**Effect size**
**Race**								
% White (count)	61 (36)	65 (63)	70 (111)	64 (34)	χ^2^ = 2.10	χ^2^ = 1.14	–	
% Multiracial (count)	15 (9)	8 (8)	11 (18)	15 (8)				
% Racial/ethnic minority (count)	24 (14)	27 (26)	19 (30)	21 (11)				
Education	3.70 (0.20)	3.25 (0.15)	4.37 (0.12)	4.72 (0.21)	*F* = 38.37^∗∗∗^	*F* = 0.09	*F* = 5.41^∗^	S.O = 0.10 Inter. = 0.02
Age (in years)	22.95 (0.65)	21.24 (0.51)	26.26 (0.40)	25.68 (0.69)	*F* = 45.26^∗∗∗^	*F* = 3.94^∗^	*F* = 0.95	S.O = 0.11 Gender = 0.01
Relationship permanence	1.41 (0.21)	1.60 (0.16)	1.08 (0.13)	1.38 (0.22)	*F* = 2.27	*F* = 1.81	*F* = 0.09	

*Effect size for chi-squared analyses is φ and effect size for ANOVA analyses is reported as partial η^2^. Only effect sizes of significant results are shown. Education was assessed using a scale of 1 = “less than a high school degree” to 8 = “professional degree (MD or JD).” Romantic relationship permanence was assessed using 0 = “single” and 5 = “almost 100% chance relationship is permanent.” ^∗^*p* < 0.05, ^∗∗∗^*p* < 0.001.*

#### Future Life Desires

Eight items were used to assess future life desires. Participants were asked to respond to the following question for a variety of life achievements, “Rate how much you DESIRE the following to describe you at the time you are 40 years old.” Participants responded to the following prompts: (1) Be a parent, (2) be married, (3) be an active part of your local community, (4) be an active part of a friendly social group, (5) obtain educational goals, (6) have a meaningful job, (7) live in ideal housing, and (8) be financially stable. Participants could respond on a scale ranging from −2 = “Very undesired” to 2 = “Very desired” with 0 = “Neither desired nor undesired.” Items were based on earlier research about future life desires in young adulthood, and we selected the age of 40 years because this age was situated near the beginning of middle adulthood (Hooker et al., 1996; Frazier and Hooker, 2006). Considered as a group, these items had adequate internal reliability, α = 0.75, and were correlated with one another, with the exception that parenthood and friendship desires which were not significantly correlated in pairwise comparisons (Table 2). Scores were averaged across items to create an overall Future Life Desire score, ranging from −2 to 2, with higher scores representing greater desires.

**TABLE 2:** Means, standard deviations, and correlation matrix of future life desires.

**Future life desires**	***M***	**SD**	**1**	**2**	**3**	**4**	**5**	**6**	**7**	**8**
1. Parenthood	0.49	1.48	1							
2. Marriage	1.25	0.99	0.52^∗∗∗^	1						
3. Community connection	0.82	0.92	0.23^∗∗∗^	0.24^∗∗∗^	1					
4. Friendships	1.38	0.82	0.02	0.21^∗∗∗^	0.35^∗∗∗^	1				
5. Education	1.48	0.90	0.18^∗∗∗^	0.27^∗∗∗^	0.29^∗∗∗^	0.26^∗∗∗^	1			
6. Meaningful career	1.77	0.67	0.22^∗∗∗^	0.42^∗∗∗^	0.23^∗∗∗^	0.39^∗∗∗^	0.49^∗∗∗^	1		
7. Ideal housing	1.55	0.77	0.19^∗∗∗^	0.32^∗∗∗^	0.27^∗∗∗^	0.25^∗∗∗^	0.35^∗∗∗^	0.56^∗∗∗^	1	
8. Financial stability	1.82	0.65	0.14^∗∗^	0.36^∗∗∗^	0.19^∗∗∗^	0.34^∗∗∗^	0.47^∗∗∗^	0.78^∗∗∗^	0.61^∗∗∗^	1
Age	24.32	5.47	−0.26^∗∗∗^	−0.21^∗∗∗^	−0.07	0.03	−0.19^∗∗∗^	−0.02	−0.01	−0.02
Current education level	4.02	1.61	−0.16^∗∗^	−0.09^†^	0.00	0.06	−0.14^∗∗^	0.03	−0.04	0.05

*^†^*p* < 0.10, ^∗^*p* < 0.05, ^∗∗^*p* < 0.01, ^∗∗∗^*p* < 0.001.*

#### Future Life Expectations

The desire items were also adapted to assess expectations. Participants were asked, “For the following statements, regardless of your desires, rate how LIKELY the following will describe you at the time you are 40 years old.” Participants responded to the same prompts from above, but these items were scaled from −2 = “Very unlikely” to 2 = “Very likely,” with 0 = “Neither likely nor unlikely.” These items showed an adequate internal reliability, α = 0.79, and were significantly correlated with one another (Table 3). All eight scores were averaged across items to create a single overall scale for Future Life Expectations, ranging from −2 to 2, with higher scores representing greater expectations.

**TABLE 3:** Means, standard deviations, and correlation matrix of future life expectations.

**Future life expectations**	***M***	**SD**	**1**	**2**	**3**	**4**	**5**	**6**	**7**	**8**
1. Parenthood	0.26	1.54	1							
2. Marriage	0.91	1.24	0.63^∗∗∗^	1						
3. Community connection	0.50	1.09	0.32^∗∗∗^	0.31^∗∗∗^	1					
4. Friendships	0.94	0.97	0.21^∗∗∗^	0.33^∗∗∗^	0.49^∗∗∗^	1				
5. Education	1.41	0.90	0.28^∗∗∗^	0.31^∗∗∗^	0.29^∗∗∗^	0.26^∗∗∗^	1			
6. Meaningful career	1.17	0.94	0.36^∗∗∗^	0.45^∗∗∗^	0.37^∗∗∗^	0.35^∗∗∗^	0.49^∗∗∗^	1		
7. Ideal housing	0.96	1.00	0.31^∗∗∗^	0.43^∗∗∗^	0.35^∗∗∗^	0.35^∗∗∗^	0.37^∗∗∗^	0.56^∗∗∗^	1	
8. Financial stability	1.09	0.96	0.26^∗∗∗^	0.36^∗∗∗^	0.29^∗∗∗^	0.26^∗∗∗^	0.42^∗∗∗^	0.59^∗∗∗^	0.63^∗∗∗^	1
Age	24.32	5.47	−0.38^∗∗∗^	−0.24^∗∗∗^	−0.06	0.03	−0.16^∗∗^	−0.01	−10^†^	−0.05
Current education level	4.02	1.61	−0.21^∗∗∗^	−0.07	0.03	0.05	0.04	0.08	−0.02	0.09

*^†^*p* < 0.10, ^∗^*p* < 0.05, ^∗∗∗^*p* < 0.001.*

### Analyses

All statistical procedures were conducted using SPSS 26. Possible differences in demographics were assessed using analyses of variance (ANOVAs), or chi-square analyses (Table 1). Heterosexual people reported younger ages than lesbian/gay individuals did, *p* < 0.001. Women reported younger ages than men did, *p* = 0.048. Heterosexual people also reported less education than did lesbian/gay people, *p* < 0.001; however, the difference in education between heterosexual men and women was less than the difference between gay men and lesbian women, *p* = 0.025 (Table 1). Because age and education varied as a function of sexual orientation, these were used as covariates in subsequent analyses. The race/ethnicity of the sample did not differ as a function of gender and sexual orientation, and no significant differences were found in relationship permanence as a function of gender and sexual orientation. Thus, race and relationship permanence were not included as covariates.

**FIGURE 1:**
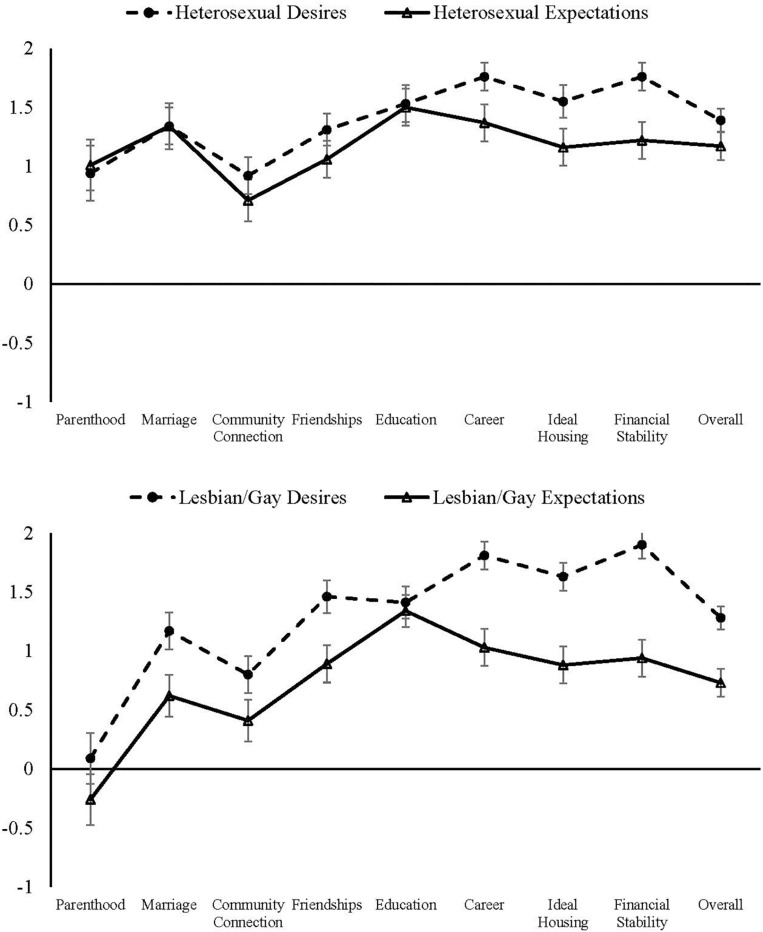
Desires and expectations for the future separated by sexual orientation. 2 represents either “Very desired” or “Very likely,” 0 represents either “Not desired nor undesired” or “Not likely nor unlikely,” and –1 represents either “Somewhat undesired” or “Somewhat unlikely.” Bars represent 95% CIs, and lines connecting outcomes are shown for visualization purposes only.

Multivariate analyses of covariance (MANCOVAs) were used to assess the differences in overall future life desires and expectations scores, and analyses of covariance (ANCOVAs) were used to assess overall scores and averaged scores as a function of gender and sexual orientation (Tables 4, 5 and Figure 1). All *post hoc* pairwise differences were assessed using the Sidak correction for multiple comparisons (Šidák, 1967).

**TABLE 4:** Future life desires as a function of gender and sexual orientation.

	**Sexual orientation (S.O)**		**Gender**					
**Future life desires *n* =**	**Heterosexual (157)**	**Gay/Lesbian (211)**	**Male** **(218)**	**Female (158)**	***F*_S.O_**	***F*_Gender_**	***F*_Interaction_**	**Partial η^2^**
Parenthood	0.94 (0.12)	0.09 (0.11)	0.65 (0.11)	0.38 (0.12)	25.06^∗∗∗^	3.04^†^	0.28	S.O = 0.07
Marriage	1.34 (0.08)	1.17 (0.08)	1.25 (0.07)	1.25 (08)	2.13	*F* < 0.01	0.50	
Community connection	0.92 (0.08)	0.80 (0.08)	0.77 0.07	0.95 0.08	1.30	3.10^†^	0.11	
Friendships	1.31 (0.07)	1.46 (0.07)	1.29 0.06	1.48 0.07	2.49	3.92^∗^	0.02	Gender = 0.01
Education	1.53 (0.08)	1.41 (0.07)	1.46 (0.07)	1.48 (0.08)	1.19	0.05	0.67	
Meaningful career	1.76 (0.06)	1.81 (0.06)	1.75 (0.05)	1.83 (0.06)	0.41	1.01	0.51	
Ideal housing	1.55 (0.07)	1.63 (0.06)	1.50 (0.06)	1.68 (0.07)	0.70	4.34^∗^	2.74^†^	Gender = 0.01
Financial stability	1.76 (0.06)	1.90 (0.06)	1.80 (0.05)	1.87 0.06	3.21^†^	0.86	0.83	
Average score	1.39 (0.05)	1.28 (0.05)	1.31 (0.04)	1.36 (0.05)	2.39	0.79	0.01	

*Results of MANCOVA are shown for the scale items preceding the results of the ANCOVA of the overall scale. Scale items are scored from −2 = “Very undesired” to 2 = “Very desired.” Means are presented above standard errors. Models used age and education as covariates. Effect sizes only shown for significant results. Multivariate results were not significant for gender or interaction effects. ^†^*p* < 0.10, ^∗^*p* < 0.05, ^∗∗∗^*p* < 0.001.*

**TABLE 5:** Future life expectations as a function of gender and sexual orientation.

	**Sexual orientation (S.O)**		**Gender**					
**Future life expectations *n* =**	**Heterosexual (157)**	**Gay/Lesbian (211)**	**Male (218)**	**Female (158)**	***F*_S.O_**	***F*_Gender_**	***F*_Interaction_**	**Partial η2**
Parenthood	1.01 (0.11)	−0.26 (0.11)	0.37 (0.10)	0.37 (0.11)	64.27^∗∗∗^	*F* < 0.01	0.39	S.O = 0.15
Marriage	1.34 (0.10)	0.62 (0.09)	0.91 (0.09)	1.06 (0.10)	26.31^∗∗∗^	1.42	0.13	S.O = 0.07
Community connection	0.71 (0.09)	0.41 (0.09)	0.47 (0.08)	0.65 (0.09)	5.05^∗^	2.06	0.42	S.O = 0.01
Friendships	1.06 (0.08)	0.89 (0.08)	0.86 (0.07)	1.09 (0.08)	2.00	4.28^∗^	0.01	Gender = 0.01
Education	1.50 (0.08)	1.34 (0.07)	1.39 (0.07)	1.46 (0.08)	2.17	0.45	0.16	
Meaningful career	1.37 (0.08)	1.03 (0.08)	1.12 (0.07)	1.27 (0.08)	9.05^∗∗^	2.17	0.08	S.O = 0.02
Ideal housing	1.16 (0.08)	0.88 (0.08)	0.95 (0.08)	1.09 (0.09)	5.73^∗^	1.57	0.90	S.O = 0.02
Financial stability	1.22 (0.08)	0.94 (0.08)	1.04 (0.71)	1.12 (0.08)	6.31^∗^	0.58	3.61^†^	S.O = 0.02
Average score	1.17 (0.06)	0.73 (0.06)	0.89 (0.05)	1.01 (0.06)	28.75^∗∗∗^	2.68	0.01	S.O = 0.07

*Results of MANCOVA are shown for the scale items preceding the results of the ANCOVA of the overall scale. Scale items are scored from −2 = “Very unlikely” to 2 = “Very likely.” Means are presented above standard errors. Models used age and education as covariates. Effect sizes only shown for significant results. Multivariate results were not significant for gender or interaction effects. ^†^*p* < 0.10, ^∗^*p* < 0.05, ^∗∗^*p* < 0.01, ^∗∗∗^*p* < 0.001.*

Chi-square analyses were then conducted to analyze the proportion of people who reported future desires that were different from expectations for each individual item as a function of sexual orientation (Table 6). Items were matched for content, i.e., parenthood expectations and parenthood desires, for comparison. Those who scored above 0.5 for desires were coded as having the desire to achieve an outcome, while those who reported <0.5 were coded as not desiring to achieve an outcome. Participants who scored >0.5 on expectations were coded as having the expectation to achieve an outcome, while those who scored <0.5 were coded as not expecting to achieve an outcome. The value 0.5 was selected because the average scores for overall desires and expectations produced values between 0 and 1, and values <0.5 would round to 0 and those >0.5 would round to 1. A more constrictive cutoff value equaling 0 was also examined, and revealed similar results as the 0.5 cutoff (Table 6). For individual items, however, both scoring methods meant that those who scored above 0 were coded as desiring or expecting outcomes.

**TABLE 6:** Desire-expectations disparity proportions as a function of sexual orientation.

	**Heterosexual (%)**	**Lesbian/Gay (%)**	**χ^2^**	**Phi**
Parenthood			82.75^∗∗∗^	0.47
Desired and expected	70^a^	26^b^		
Not desired and not expected	20^a^	50^b^		
Desired, but not expected	3^a^	20^b^		
Expected, but not desired	7^a^	4^a^		
Marriage			57.49^∗∗∗^	0.40
Desired and expected	80^a^	48^b^		
Not desired and not expected	8^a^	22^b^		
Desired, but not expected	3^a^	26^b^		
Expected, but not desired	8^a^	4^a^		
Community connection			7.41^†^	0.14
Desired and expected	59^a^	44^b^		
Not desired and not expected	22^a^	31^a^		
Desired, but not expected	14^a^	18^a^		
Expected, but not desired	5^a^	7^a^		
Friendships			8.56^∗^	0.15
Desired and expected	75^a^	69^a^		
Not desired and not expected	11^a^	8^a^		
Desired, but not expected	10^a^	21^b^		
Expected, but not desired	4^a^	2^a^		
Education			13.49^∗∗^	0.19
Desired and expected	89^a^	73^b^		
Not desired and not expected	3^a^	9^b^		
Desired, but not expected	4^a^	8^a^		
Expected, but not desired	5^a^	9^a^		
Meaningful career			16.43^∗∗∗^	0.21
Desired and expected	87^a^	76^b^		
Not desired and not expected	3^a^	0^b^		
Desired, but not expected	9^a^	22^b^		
Expected, but not desired	2^a^	1^a^		
Ideal housing			12.59^∗∗^	0.19
Desired and expected	83^a^	68^b^		
Not desired and not expected	5^a^	5^a^		
Desired, but not expected	10^a^	23^b^		
Expected, but not desired	2^a^	4^a^		
Financial stability			11.86^∗∗^	0.18
Desired and expected	82^a^	73^a^		
Not desired and not expected	3^a^	0^b^		
Desired, but not expected	13^a^	25^b^		
Expected, but not desired	2^a^	2^a^		
Overall future aspirations^1^			24.49^∗∗∗^	0.25
Desired and expected	89^a^	68^b^		
Not desired and not expected	3^a^	4^a^		
Desired, but not expected	7^a^	26^b^		
Expected, but not desired	2^a^	2^a^		
Overall future aspirations^2^			27.75^∗∗∗^	0.26
Desired and expected	94^a^	83^b^		
Not desired and not expected	3^a^	0^b^		
Desired, but not expected	2^a^	16^b^		
Expected, but not desired	2^a^	1^a^		

*Fisher’s exact test used when at least one cell had an expected count lower than five participants. Different letters denote significant differences. ^1^Overall future aspirations using 0.5 as a cutoff value. ^2^Overall future aspirations using 0 as a cutoff value. ^†^*p* < 0.10, ^∗^*p* < 0.05, ^∗∗^*p* < 0.01, ^∗∗∗^*p* < 0.001.*

Using this coding scheme, four groups emerged: (1) those who desired and expected to achieve an outcome, (2) those who did not desire and did not expect to achieve an outcome, (3) those who desired, but did not expect to achieve an outcome, and (4) those who expected, but did not desire to achieve an outcome. Differences in the proportion of individuals in these groups were examined as a function of sexual orientation. Fisher’s exact tests were used when at least one cell had an expected count lower than five participants.

### Preliminary Analyses

Age and education were significantly correlated with desires and expectations for the future (Tables 2, 3). Older participants reported fewer desires for parenthood, marriage, and education, *p* < 0.001 for all. Higher education levels were associated with lower desires for parenthood, *p* = 0.002, and lower desires to obtain further educational achievement, *p* = 0.006. Older participants also reported lower expectations for parenthood, *p* < 0.001, marriage, *p* < 0.001, and educational achievement, *p* = 0.003. Having more education was also associated with lower expectations for parenthood, *p* < 0.001.

## Results

We report the results in three sections. We report first on desires for the future, then on expectations for the future, and finally on gaps between future desires and expectations.

### Desires for the Future

Multivariate analyses found a significant result for sexual orientation when assessing the items about future desires, Wilks’ Lambda = 0.90, *F*(8,355) = 4.76, *p* < 0.001, partial η^2^ = 0.10 (Table 4). Univariate analyses revealed only one difference as a function of sexual orientation, and that was for parenthood desires, *F*(1,362) = 25.06, *p* < 0.001. As expected, lesbian/gay individuals reported less desire to pursue parenthood than did heterosexual participants, *p* < 0.001. However, no univariate difference in the average score for future desires was found as a function of gender or sexual orientation. Multivariate tests for gender and the interactions between gender and sexual orientation did not show significant findings. Univariate results showed that women reported greater desires for friendships, *F*(1,362) = 3.92, *p* = 0.048, and to live in ideal housing, *F*(1,362) = 4.34, *p* = 0.038, than did men, but these results should be viewed with caution, in light of non-significant multivariate findings.

### Expectations for the Future

Multivariate analyses revealed a significant main effect for sexual orientation on future expectations, Wilks’ Lambda = 0.85, *F*(8,355) = 8.10, *p* < 0.001, partial η^2^ = 0.15 (Table 5). Univariate analyses revealed differences as a function of sexual orientation in expectations for parenthood, *F*(1,362) = 64.27, *p* < 0.001; marriage, *F*(1,362) = 26.31, *p* < 0.001; community connection, *F*(1,362) = 5.05, *p* = 0.025; meaningful career, *F*(1,362) = 9.05, *p* = 0.003; housing, *F*(1,362) = 5.73, *p* = 0.017; and financial stability, *F*(1,362) = 6.31, *p* = 0.012. Lesbian/gay participants reported lower expectations for parenthood, *p* < 0.001; marriage, *p* < 0.001; community connection, *p* = 0.025; meaningful careers, *p* = 0.003; housing, *p* = 0.017; and financial stability, *p* = 0.012, than did heterosexual individuals. When assessing the average score for future expectations, lesbian/gay individuals reported lower future expectations than did heterosexual individuals, *F*(1,362) = 28.75, *p* < 0.001. Multivariate tests found no significant effects for gender or for the interactions between gender and sexual orientation. Univariate analyses found that women expected to have friendships more than did men, *F*(1,362) = 4.34, *p* = 0.039, but this result should be viewed with caution, in light of the non-significant multivariate test.

### Gaps Between Desires and Expectations

We also found significant gaps between desires and expectations for the future as a function of sexual orientation (Table 6 and Figure 1). Chi-square analyses indicated significant gaps in aspirations for the future overall, χ^2^ = 24.49, *p* < 0.001, φ = 0.25. More specifically, differences between desires and expectations emerged for parenthood, χ^2^ = 2.75, *p* < 0.001, φ = 0.47; marriage, χ^2^ = 57.49, *p* < 0.001, φ = 0.40; friendships, χ^2^ = 8.56, *p* = 0.033, φ = 0.15; education, χ^2^ = 13.49, *p* = 0.004, φ = 0.19; meaningful career, χ^2^ = 16.43, *p* < 0.001, φ = 0.21; ideal housing, χ^2^ = 12.59, *p* = 0.006, φ = 0.19; and financial stability, χ^2^ = 11.86, *p* = 0.005, φ = 0.18.

A greater proportion of lesbian and gay individuals (26%) than heterosexual individuals (7%) reported desiring overall future outcomes, but not expecting to achieve them, *p* < 0.001. When assessing individual aspirations for the future, a greater proportion of lesbian and gay individuals than heterosexual individuals reported desiring, but not expecting to achieve parenthood, *p* < 0.001; marriage, *p* < 0.001; friendships, *p* = 0.007; meaningful careers, *p* < 0.001; ideal housing, *p* = 0.002; and financial stability, *p* = 0.008.

A greater proportion of heterosexual individuals (89%) than lesbian and gay individuals (68%) reported that their expectations for overall future outcomes matched their desires, *p* < 0.001. More heterosexual individuals than lesbian and gay individuals reported both desires and expectations for parenthood, *p* < 0.001, marriage, *p* < 0.001, education, *p* < 0.001, meaningful careers, *p* = 0.014, and ideal housing, *p* = 0.001.

Moreover, a greater proportion of lesbian and gay individuals than heterosexual individuals reported neither desiring nor expecting parenthood, *p* < 0.001; marriage, *p* < 0.001; or educational achievement, *p* = 0.019. There were no significant differences in the proportions of those who reported expectations for the future, but not desires as a function of sexual orientation.

## Discussion

This study examined how lesbian, gay, and heterosexual young adults envision their futures. Much of the existing research focused on disparities in aspirations about future family formation (Patterson and Riskind, 2010; Riskind and Patterson, 2010; Baiocco and Laghi, 2013; Gato et al., 2017; Riskind and Tornello, 2017; Simon et al., 2018; Jeffries et al., 2019; Tate et al., 2019). Whether or not such disparities extended to other aspirations for the future has received less study. We explored a variety of desires and expectations for the future among lesbian, gay, and heterosexual young adults to assess potential disparities as a function of sexual orientation.

As expected, lesbian and gay individuals were more likely than heterosexual individuals to report lower desire and fewer expectations for parenthood (Baiocco and Laghi, 2013; Gato et al., 2017; Riskind and Tornello, 2017; Simon et al., 2018; Tate et al., 2019). In addition, lesbian and gay individuals were also more likely than their heterosexual counterparts to report lower expectations for marriage, community connection, meaningful employment, housing, and financial stability. These findings are consistent with our prediction that negative expectations for the future encompass multiple aspects of life among lesbian and gay young adults.

Apart from parenthood, desires for the future did not differ as a function of gender or sexual orientation. Lesbian and gay young adults hoped lead lives very much like those envisioned by their heterosexual counterparts. Previous research has found that lesbian and gay youth envision family formation very much as do heterosexual individuals (D’Augelli et al., 2008). Our study extended this finding by showing that lesbian and gay young adults also hope for other life outcomes, such as financial stability, that are similar to those that heterosexual individuals envision.

As expected, lesbian and gay individuals were more likely than heterosexual peers to want future outcomes that they did not expect to achieve. A disparity between desires and expectations for the future has been found repeatedly for aspects of family formation (Shenkman, 2012; Riskind and Tornello, 2017), but our results showed that disparities in desires and expectations for the future are much more widespread across outcomes. The largest effect sizes found for disparities between desires and expectations were in marriage and parenthood, but significant disparities also emerged in other domains.

These findings have significant implications for our understanding of family formation. Much of the work on parenthood aspirations has focused only on parenthood (Baiocco and Laghi, 2013; Gato et al., 2017; Riskind and Tornello, 2017; Leal et al., 2019; Tate et al., 2019). Our findings suggest that disparities in family formation may be only one part of a larger issue. Research has shown that envisioning a hoped-for future that one believes to be out of reach has negative effects on mental health (Frazier and Hooker, 2006). The current results reveal that lesbian and gay young adults are far more likely than their heterosexual peers to hope for futures that they do not think they can achieve. Thus, our findings may underlie some known health disparities as a function of sexual orientation (National Institutes of Health, 2016; Patterson et al., 2018; Tate and Patterson, 2019b). Future work should examine the possible associations of such discrepancies with mental and physical health.

Moreover, the connections between disparities in parenthood aspirations and other future life aspirations as a function of sexual orientation need to be examined. For instance, differences in social networks seem to explain part of the disparities between lesbian/gay and heterosexual parenthood aspirations (Simon et al., 2018; Tate and Patterson, 2019a; Tate et al., 2019). In addition, disparities in aspirations about marriage may also help to explain part of the disparities in aspirations about parenthood. Parenthood tends to be normalized for heterosexual people within marriage (Ashburn-Nardo, 2017), but whether or not this is true for sexual minorities needs more investigation. Future work should examine how differences in other future aspirations could help account for differences in parenthood aspirations as a function of sexual orientation.

While this research had strengths and produced novel findings, it was not without limitations. For instance, while desires and expectations for the future were examined, intentions to pursue these future life outcomes were not studied here. Also, beliefs of those with plurisexual identities could not be examined due to small sample sizes. In addition, and also due to sample size constraints, comparisons among racial groups were not possible. Data were collected over social media, so the degree to which these results are representative of the population of lesbian, gay, or heterosexual individuals could not be assessed. Also, the study employed a cross-sectional design, so causal influences could not be identified. Even so, the study produced valuable new information.

In all, for lesbian and gay young adults, low parenthood aspirations were part of a general pattern of low expectations, though not reduced desires, across a number of life domains. Lesbian, gay, and heterosexual young adults seem to be hoping for similar futures, but expecting vastly different outcomes from one another. These findings thus have significant implications not only in understanding the LGBT family life course, but also for work on mental and physical health disparities that impact the lives of lesbian and gay people.

## Data Availability Statement

The datasets generated for this study will not be made publicly available. Requests for datasets should be sent to the first author.

## Ethics Statement

The studies involving human participants were reviewed and approved by the University of Virginia Institutional Review Board. The patients/participants provided their written informed consent to participate in this study.

## Author Contributions

Both authors listed have made a substantial, direct and intellectual contribution to the work, and approved it for publication.

## Conflict of Interest

The authors declare that the research was conducted in the absence of any commercial or financial relationships that could be construed as a potential conflict of interest.

## References

[B1] Ashburn-Nardo L. (2017). Parenthood as a moral imperative? moral outrage and the stigmatization of voluntarily childfree women and men. *Sex Roles* 76 393–401. 10.1007/s11199-016-0606-1

[B2] Baiocco R. Laghi F. (2013). Sexual orientation and the desires and intentions to become parents. *J. Fam. Stud.* 9 90–98. 10.5172/jfs.2013.19.1.90

[B3] Berkowitz D. Marsiglio W. (2007). Gay men negotiating procreative, father, and family identities. *J. Marriage Fam.* 69 366–381. 10.1111/j.1741-3737.2007.00371.x

[B4] Blake L. Carone N. Raffanello E. Slutsky J. Ehrhardt A. A. Golombok S. (2017). Gay fathers’ motivations for and feelings about surrogacy as a path to parenthood. *Hum. Reprod.* 32 1–8. 10.1093/humrep/dex026 28333218 PMC5400050

[B5] D’Augelli A. R. Rendina H. J. Sinclair K. O. (2008). Gay and lesbian youth want long-term couple relationships and raising children. *J. LGBT Issues Couns.* 1 77–98.

[B6] Frazier L. D. Hooker K. (2006). “Possible selves in adult development: linking theory and research,” in *Possible Selves: Theory, Research and Application*, eds Dunkel C. K. Kerpelman J. (New York, NY: Nova Science Publishers), 41–59.

[B7] Gato J. Santos S. Fontaine A. M. (2017). To have or not to have children? that is the question. factors influencing parental decisions among lesbians and gay men. *Sex Res. Soc. Policy* 14 310–323. 10.1007/s13178-016-0268-3 29237004

[B8] Goldberg A. E. Downing J. B. Moyer A. M. (2012). Why parenthood, and why now? gay men’s motivations for pursuing parenthood. *Interdiscip. J. Appl. Fam. Stud.* 61 157–174. 10.1111/j.1741-3729.2011.00687.x 22563135 PMC3341136

[B9] Goldberg A. E. Downing J. B. Sauck C. C. (2007). Choices, challenges, and tensions: perspectives of lesbian prospective adoptive parents. *Adopt. Q.* 10 33–64. 10.1300/J145v10n02_02

[B10] Hooker K. Fiese B. H. Jenkins L. Morfei M. Z. Schwagler J. (1996). Possible selves among parents of infants and preschoolers. *Develop. Psychol.* 32 542–550. 10.1037/0012-1649.32.3.542

[B11] Jeffries W. L. Marsiglio W. Tunalilar O. Berkowitz D. (2019). Fatherhood desires and being bothered by future childlessness among U.S. Gay, bisexual, and heterosexual men—United States, 2002–2015. *J. GLBT Fam. Stud.* 1–16. 10.1080/1550428X.2019.1652876

[B12] Leal D. Gato J. Tasker F. (2019). Prospective parenting: sexual identity and intercultural trajectories. *Cult. Health Sex* 21 757–773. 10.1080/13691058.2018.1515987 30355177

[B13] National Coalition of Anti-Violence Programs (2018). *A Crisis of Hate: A Report on Homicides Against Lesbian, Gay, Bisexual and Transgender People.* Available at: http://avp.org/wp-content/uploads/2018/01/a-crisis-of-hate-january-release-12218.pdf (accessed September 1, 2019).

[B14] National Institutes of Health (2016). *Sexual and Gender Minorities Formally Designated as a Health Disparity Population for Research Purposes.* Available at: https://www.nimhd.nih.gov/about/directors-corner/message.html (accessed September 1, 2019).

[B15] Patterson C. J. Riskind R. G. (2010). To be a parent: issues in family formation among gay and lesbian adults. *J. GLBT Fam. Stud.* 6 326–340. 10.1080/1550428X.2010.490902 29135378

[B16] Patterson C. J. Tate D. P. Sumontha J. Xu R. (2018). Sleep difficulties among sexual minority adults: associations with family relationship problems. *Psychol. Sex Orientat. Gend. Divers.* 5 109–116. 10.1037/sgd0000264

[B17] Quartey N. (2018). *Corporate Activism in the Age of LGBT Equality: The Promise and Limitations of the Modern Executive Champion on LGBT Rights.* Available at: https://search.proquest.com/docview/2128056976?accountid=14678 (accessed September 1, 2019).

[B18] Rabelo V. C. Cortina L. M. (2014). Two sides of the same coin: gender harassment and heterosexist harassment in LGBQ work lives. *Law Hum. Behav.* 38 378–391. 10.1037/lhb0000087 24933169

[B19] Riskind R. G. Patterson C. J. (2010). Parenting intentions and desires among childless lesbian, gay, and heterosexual individuals. *J. Fam. Psychol.* 24 78–81. 10.1037/a0017941 20175611

[B20] Riskind R. G. Tornello S. L. (2017). Sexual orientation and future parenthood in a 2011–2013 nationally representative United States sample. *J. Fam. Psychol.* 31 792–798. 10.1037/fam0000316 28368202

[B21] Robinson M. A. Brewster M. E. (2014). Motivations for fatherhood: examining internalized heterosexism and gender-role conflict with childless gay and bisexual men. *Psychol. Men Masc.* 15 49–59. 10.1037/a0031142

[B22] Scandurra C. Bacchini D. Esposito C. Bochicchio V. Valerio P. Amodeo A. L. (2019). The influence of minority stress. gender, and legalization of civil unions on parenting desire and intention in lesbian women and gay men: implications for social policy and clinical practice. *J. GLBT Fam. Stud.* 15 76–100. 10.1080/1550428X.2017.1410460

[B23] Shenkman G. (2012). The gap between fatherhood and couplehood desires among Israeli gay men and estimations of their likelihood. *J. Fam. Psychol.* 26 828–832. 10.1037/a0029471 22888780

[B24] Shenkman G. Bos H. Kogan S. (2019). Attachment avoidance and parenthood desires in gay men and lesbians and their heterosexual counterparts. *J. Reprod. Infant Psychol.* 37 344–357. 10.1080/02646838.2019.1578872 30773903

[B25] Šidák Z. K. (1967). Rectangular confidence regions for the means of multivariate normal distributions. *J. Am. Stat. Assoc.* 62 626–633. 10.1080/01621459.1967.10482935

[B26] Simon K. A. Tornello S. L. Farr R. H. Bos H. M. W. (2018). Envisioning future parenthood among bisexual, lesbian, and heterosexual women. *Psychol. Sex Orientat. Gen. Divers.* 5 253–259. 10.1037/sgd0000267

[B27] Tate D. P. Patterson C. J. (2019a). Sexual minority women’s attitudes toward infants, children, and parenthood. *J. Lesbian Stud.* 23 464–475. 10.1080/10894160.2019.1629807 31218942

[B28] Tate D. P. Patterson C. J. (2019b). Sexual orientation, relationships with parents, stress, and depressive symptoms among adults. *J. GLBT Fam. Stud.* 15 256–271. 10.1080/1550428X.2018.1486263

[B29] Tate D. P. Patterson C. J. Levy A. J. (2019). Predictors of parenting intentions among childless lesbian, gay, and heterosexual adults. *J. Fam. Psychol.* 33 194–202. 10.1037/fam0000499 30589288

